# The Interplay of Heart Failure and Lung Disease: Clinical Correlations, Mechanisms, and Therapeutic Implications

**DOI:** 10.70322/jrbtm.2024.10020

**Published:** 2024-12-02

**Authors:** Salma Ahmad, Ayman Isbatan, Sunny Chen, Steven M. Dudek, Richard D. Minshall, Jiwang Chen

**Affiliations:** 1 Cardiovascular Research Center, University of Illinois at Chicago, Chicago, IL 60612, USA; 2 Division of Pulmonary, Critical Care Medicine, Sleep and Allergy, Department of Medicine, University of Illinois at Chicago, Chicago, IL 60612, USA; 3 Department of Anesthesiology, University of Illinois at Chicago, Chicago, IL 60612, USA

**Keywords:** Chronic heart failure (CHF), Acute heart failure (AHF), Lung dysfunction, Chronic systemic inflammation, Pressure overload

## Abstract

Heart failure (HF) is a common clinical syndrome marked by reduced cardiac output, elevated intracardiac pressures, and heart dysfunction. Chronic HF (CHF) is a syndrome characterized by a lack of blood flow and impaired pumping ability to the heart over time, while acute HF (AHF) arises suddenly due to incidents like myocardial infarction or cardiac arrest. HF has a significant impact on pulmonary health and function, leading to conditions such as pulmonary edema and restrictive lung patterns. Clinical evidence highlights the bidirectional relationship between HF and lung dysfunction. Declining lung function serves as a predictor for HF progression and severity, while HF contributes to worsening lung health. Animal models that induce HF through surgical methods further demonstrate the connection between heart and lung pathology. The main mechanisms linking HF and lung dysfunction are pressure overload and chronic systemic inflammation, with changes in the extracellular matrix (ECM) also playing a role. Additionally, environmental factors like air pollution exacerbate lung inflammation, increasing the risk of both HF and chronic obstructive pulmonary disease (COPD) incidence. Combined treatment approaches involving pharmaceutical drugs such as statins, Angiotensin-converting enzyme (ACE) inhibitors, and Angiotensin receptor blockers (ARBs) may benefit by reducing inflammation. This review will explore the complex interplay between HF and lung function, emphasizing their interconnected pathophysiology and potential integrated treatment strategies.

## 1. Introduction

Heart failure (HF) is defined as a condition of reduced cardiac output, elevated intracardiac pressures, and general heart dysfunction due to structural and functional abnormality of the heart [1]. HF is a clinical syndrome that affects around 160 million people worldwide [1] and is characterized by symptoms such as tissue swelling, shortness of breath, and fatigue [2]. Chronic HF (CHF), defined by the American College of Cardiology (ACC) and the American Heart Association (AHA), is a syndrome that results from any structural or functional impairment of ventricular filling or ejection of blood [3]. CHF is one of the most common health conditions in the U.S, with one of the highest morbidity and mortality rates [4].

Coronary artery disease (CAD) is the leading cause of CHF [5]: as of October 2021, approximately 360,000 people in the U.S die each year due to CAD-induced HF [6]. While common risk factors like smoking, age, and systemic inflammation contribute to both coronary artery disease (CAD)-induced CHF and chronic obstructive pulmonary disease (COPD) [7,8], the heart-lung interaction introduces additional layers of physiological complexity. People with COPD, for example, have a significantly higher risk of developing CAD due to the added strain on the heart, which can then subsequently develop into CHF [9]. This interaction, particularly through mechanisms like pressure overload and chronic inflammation, increases the prevalence and worsens outcomes in patients with both conditions [10,11]. Other common etiologies of CHF include hypertension and valvular heart disease [4,12].

Acute HF (AHF) is generally defined as a life-threatening clinical syndrome with rapidly developing or worsening symptoms and signs of typical HF requiring emergent treatment [13]. AHF is any heart failure that develops suddenly, such as after a heart attack, cardiac arrest, or with arrhythmia (irregular heartbeat). More than 80% of hospitalized AHF cases occur in patients over the age of 65 years [14]. In most cases, AHF arises due to deterioration in patients with a previous diagnosis of CHF and often as a result of a clear trigger such as arrhythmia [15]. The most common etiologies include myocardial infarction, a decreased or complete cessation of blood flow to the myocardium [16], and cardiac arrest, a sudden electrical malfunction in the heart that causes it to stop beating entirely [17].

**Figure 1.**
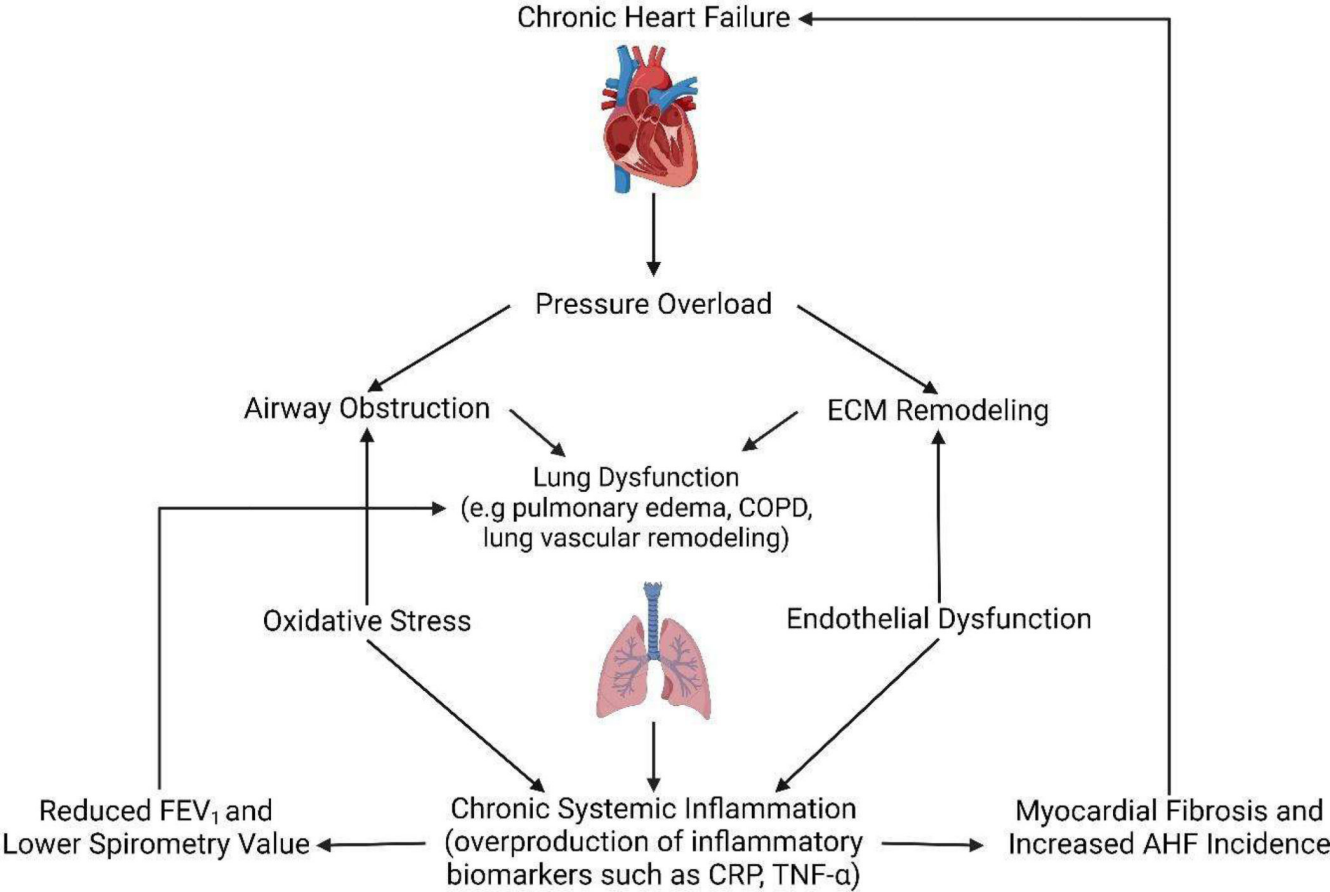
Mechanisms linking Heart Failure and Lung Dysfunction. CHF (chronic heart failure) initiates increased pressure within the heart, leading to systemic pressure overload. This pressure overload results in increased resistance in the airways, contributing to obstruction and reduced airflow, and induces changes in the ECM (extracellular matrix) of lung tissues, leading to remodeling and structural alterations. Airway obstruction contributes to lung dysfunction, including pulmonary edema and COPD, while ECM remodeling exacerbates lung dysfunction by impacting lung tissue function. Lung dysfunction triggers chronic systemic inflammation, characterized by an overproduction of inflammatory biomarkers such as CRP (C-reactive protein) and TNF-α. Chemical imbalances triggered by oxidative stress and endothelial dysfunction further exacerbate chronic systemic inflammation. Oxidative stress contributes to airway obstruction, while endothelial dysfunction exacerbates ECM remodeling. Chronic systemic inflammation leads to reduced forced expiratory volume in one second (FEV1) and triggers myocardial fibrosis and increased AHF incidence, creating a feedback loop by further contributing to lung dysfunction and CHF. This figure was created with BioRender.com.

It has long been established that HF is linked to lung dysfunction. The cardiovascular and respiratory systems are closely interconnected in physicality and function (Figure 1). The heart pumps oxygenated blood throughout the body, while the lungs reoxygenate the blood by exchanging oxygen and carbon dioxide. Deoxygenated blood from the body is collected in the heart’s right atrium, pumped through the pulmonary arteries to the lungs for reoxygenation, and then returned to the heart to repeat the cycle [18]. Due to this interconnection, HF often leads to respiratory issues like restrictive patterns and increased lung fluids. Abnormalities and restrictions in lung function are frequently observed in patients with CHF in the absence of respiratory disease. Specifically, cardiac enlargement and filling leads to reduction in alveolar space and limits the ability of the lungs to fill adequately, leading to worse disease prognosis as airway obstruction causes inflammation and other complications [19]. In the case of AHF, which can be dramatic and rapid in onset, flash pulmonary edema is a common symptom: when heart failure worsens suddenly, pulmonary fluid homeostasis is disturbed, causing fluid to leak into the lungs and lead to pulmonary edema [20].

This review aims to deepen the understanding of the intricate connections between heart and lung pathologies by presenting both clinical and experimental evidence. The mechanisms underlying the interaction between these two systems will be thoroughly examined, providing insight into how heart failure can exacerbate pulmonary complications. Additionally, this review will explore future treatment and management strategies, with the goal of developing more effective therapies for heart failure patients who experience pulmonary complications.

## 2. Clinical Evidence

There is substantial clinical evidence showing a strong association between HF and a decline in lung function, highlighting the complex relationship between these two vital systems. Pulmonary function and efficiency are often a predictor of mortality in patients with heart disease [21]. Lung function determines airway flow, lung volume and capacity, and how efficiently the lungs oxygenate the blood. The anatomical and physiological structure of the lungs is closely connected to the heart and blood vessels, so any significant issues with lung function can impair cardiovascular health [22].

In the case of CHF (CHF), numerous patient and population studies have been done that recognize the link between CHF and lung dysfunction. The findings and implications of these studies are presented in Table 1. For example, one study conducted by a Cardiovascular Health Study research group found that chronic obstructive pulmonary disease (COPD) had a greater prevalence in patients with CHF (20%) than in the general population (13%) [22]. Another population meta-analysis reported the prevalence of COPD ranged from 11 to 52% in North American patients with CHF, and from 9 to 41% in European cohorts, with increased prevalence in recent studies [23].

There is also substantial evidence suggesting that declining lung function indicates a higher risk of developing HF. A community-based study of approximately 10,000 participants followed for 17 years demonstrated that a rapid decline in lung function is associated with a higher risk of cardiovascular disease, independent of other established cardiovascular risk factors. “Rapid decline” was defined as the greatest quartile of decline in the forced expiratory volume of the lungs in 1 second (FEV_1_) over a mean of approximately 3 years [24]. Furthermore, a meta-analysis of 176 references found that patients with COPD were 95% more likely to be diagnosed with cardiovascular disease, including a 2–5 times higher risk of ischemia, cardiac dysrhythmia, and general CHF [25]. The implications of this study, as shown in Table 1, demonstrate that patients with lung dysfunction are more likely than healthy patients to develop cardiovascular diseases such as CHF. Another systematic review comprising a total of almost 84,000 participants across 12 studies found that pulmonary function was the best and most accurate predictor of cardiovascular mortality. This study, which also used FEV_1_ as an indicator of lung function and health, found that individuals in the lowest quintile demonstrated a 75% increase in the risk for cardiovascular mortality compared with those in the highest quintile [26]. Looking specifically at the effects of restrictive lung function on the cardiovascular system, one patient study established a strong association between ischemic heart disease and impaired lung function–specifically, an increasing prevalence of heart disease was associated with increased COPD severity as well as high proportions of HF among subjects with restrictive lung function [27]. CHF and COPD also have shared risk factors, such as smoking, hypertension, and systemic inflammation. These factors contribute to the high prevalence of CAD among patients with either CHF or COPD, as various studies have reported a strong link between the occurrence of COPD and the presence of CAD. Systemic inflammation and pressure overload—mechanisms detailed in subsequent sections–create a feedback loop intensifying disease severity in patients with CAD-induced CHF and concurrent COPD [9,28].

Further evidence supports the idea of a bidirectional relationship between AHF and lung function: as AHF worsens pulmonary function, pulmonary dysfunction increases the risk for AHF. The European Society of Cardiology categorizes AHF into six stages based on symptoms and severity. Stage three is characterized by pulmonary edema, rapid respiratory distress, lung crackles, and a drop in oxygen levels [29]. COPD, characterized by systemic inflammation, can contribute to adverse and incident cardiovascular events, such as myocardial infarction [30]. One study found a significant association between restrictive lung function impairment and incident cardiovascular issues, or AHF, defined as myocardial infarction, stroke, or transient ischemic attacks. COPD prevalence significantly increased the hazard ratios for cardiovascular disease in another study [31]. Acute decompensated heart failure (ADHF), a common and fatal form of AHF, causes acute respiratory distress due to rapid fluid buildup within the interstitial and alveolar spaces in the lungs, leading to respiratory decompensation and dyspnea [32]. In one study, myocardial infarction is named as the main cause of pulmonary edema in patients or the accumulation of excessive fluid in the alveolar walls and alveolar spaces of the lungs. Increased pulmonary venous blood pressure leads to increased lung capillary pressure, leading to pulmonary edema [20]. Aortic stenosis, another cause of AHF, is a condition where the aortic valve narrows, causing increased pressure in the heart. Aortic stenosis is proven to cause exertional dyspnea by narrowing the airways [33].

Sleep-disordered breathing (SDB) is another critical aspect of the relationship between lung function and cardiovascular health. Conditions such as obstructive sleep apnea (OSA), a sleep-related breathing disorder that occurs when the muscles in the upper throat relax during sleep, narrowing the airway and interrupting breathing, are common in patients with CHF. One analysis of approximately 7000 patients with stable CHF receiving optimal medical care found that moderate-to-severe SDB was prevalent in 46% of patients [34]. Untreated severe OSA was linked to a three times higher risk of both fatal and nonfatal cardiovascular diseases, such as stroke and heart attack, compared to people without SDB [35].

In summary, the published evidence to date (Table 1) overwhelmingly underlines the link between lung and heart function, as lung dysfunction can cause and worsen HF, and vice versa.

## 3. Preclinical Evidence

There has also been extensive research on the association between lung and heart function in animal trials, most commonly exploring the impact of induced HF. The studies and experiments discussed in this review are presented in Table 2. Animal models of HF are essential in developing treatment strategies and understanding the pathophysiology of HF [36].

One of the most common animal surgical models used to study CHF is Transverse Aortic Constriction (TAC), also known as aortic banding. In the TAC model, a permanent constriction is made around the transverse aorta that connects the ascending and descending aortas of the heart. Initially, TAC causes compensated hypertrophy of the left ventricle, which temporarily enhances contractility and the ability to manage pressure overload. Over time, however, hypertrophy begins to fail, resulting in cardiac dilatation, a condition where the heart’s ventricles stretch and enlarge, ultimately leading to HF [37,38].

Multiple studies discuss TAC induced HF in mice and its effects on lung function. One study finds that chronic TAC in mice causes significant pulmonary fibrosis, or scarring of lung tissue, lung remodeling, and group 2 pulmonary hypertension. TAC-induced left ventricular dysfunction increases lung weight, pulmonary hypertension, and right ventricular (RV) hypertrophy. Chronic left ventricular (LV) failure leads to lung congestion, increased lung weight, and group 2 pulmonary hypertension. This serious chronic condition occurs when blood pressure in the pulmonary vasculature is higher than normal. The study establishes a link between left ventricle failure and lung disease, showing that TAC-induced HF in mice leads to significant lung remodeling and pulmonary hypertension [39]. TAC is also used in larger animals such as swine: in one study, female Ossabaw pigs were fed a Western diet for 10 months and had aortic banding to create chronic pressure overload for 6 months. This model was recognized by the National Heart, Lung, and Blood Institute to study CHF with a preserved ejection fraction. In this condition, the myocardium is thickened and stiff despite normal pumping ability. The pigs showed typical signs of HF, including increased lung weight and pulmonary hypertension [40].

Additionally, multiple studies discuss myocardial infarction, a form of AHF, in mice and its effect on lung function. In one study, MI was induced in mice by permanently ligating the left anterior descending coronary artery (LAD), the largest coronary artery in the heart, supplying oxygenated blood to the front of the left side of the heart. Ligating LAD results in significant reduction or complete cessation of blood flow to the area of the heart muscle (myocardium) it supplies, inducing myocardial infarction. The MI mice exhibited lung weight increase at around 4 weeks post-operation. 60% of the MI mice developed pleural effusion or fluid buildup in the tissue that lines the cavity between the lungs and chest area. Pulmonary edema, defined in the experiment as a significant increase in fluid retention in the lungs occurred in many of the mice due to post-MI HF [41].

However, animal trials are limited in their real-world application due to inherent physiological differences between animals and humans, and a discrepancy between the clinical conditions in human and animal models [42]. Still, the use of animal models has been crucial in the development of new clinical treatments and therapies for HF. One of the most significant accomplishments emerging from extensive animal trials and modeling was the development of β–adrenergic receptor antagonists, or beta-blockers, a class of drugs that reduce stress on the heart and blood vessels by blocking the effects of adrenaline [43,44]. Animal trials continue to be instrumental in demonstrating the connection between HF and lung dysfunction, as outlined by the implications of the above studies in Table 2.

## 4. Mechanisms

Perhaps the most well-known mechanism by which HF causes lung dysfunction is through pressure overload. CHF causes congestion and increased heart size due to an inefficiency in pumping blood, pushing blood back into the vessels that carry oxygenated blood into the lungs. As the pressure in these blood vessels increases, the fluid pushes into the lungs’ alveoli, reducing normal oxygen movement and causing high pulmonary pressures. This fluid buildup is also known as pulmonary edema, promoting airway obstruction, loss of lung volume, and impaired gas exchange [45]. Pressure overload as a mechanism has been observed to be bidirectional as well: in conditions such as right ventricular hypertrophy, abnormal enlargement of the ventricular muscle mass occurs in response to chronic pressure overload caused by chronic, severe lung disease [46]. Furthermore, pressure overload has been observed to trigger an inflammatory response in the myocardium [47], as demonstrated in the signal pathway in Figure 1.

Chronic systemic inflammation is another widely accepted and promising mechanism that links lung dysfunction and HF [48]. Chronic systemic inflammation is defined as the condition of the immune system when it is constantly activated, triggering an overreaction of inflammatory biomarkers [49]. The relationship between inflammation and heart failure (HF) is bidirectional: inflammation worsens HF, and HF, in turn, promotes inflammation. In diastolic HF or heart failure with preserved ejection fraction (HFpEF), inflammation is thought to start from a proinflammatory state of the body that arises from HFpEF, affecting the heart’s blood vessels and leading the heart muscle walls to become thicker [50]. In systolic HF or heart failure with reduced ejection fraction (HFrEF), inflammation typically results from direct damage to heart muscle cells arising from AHF, such as myocardial infarction or ischemia [51]. In both HFpEF and HFrEF, increased concentrations of proinflammatory biomarkers lead to worsened clinical outcomes [52].

The mechanism by which HF-induced inflammation leads to lung dysfunction is characterized by the inverse pathway between the systemic inflammatory marker CRP and the forced expiratory volume of the lungs in one second (FEV1) [53]. CRP, an acute-phase inflammatory protein primarily synthesized in the liver, shows elevated levels of production and expression in areas affected by inflammation [54]. CRP has consistently been found to be associated with excess mortality in cardiovascular disease and stroke and is often linked specifically with CHF, aortic valve disease, and myocardial infarction [55]. Recent research has linked restrictive lung disease to elevated CRP levels, as increased systemic inflammation has been shown to correlate with reduced spirometry-based lung function [56]. In one survey study that evaluated approximately 7000 adults under the age 50, airflow obstruction was found to be an important risk factor for cardiac injury and incident AHF: due to elevated levels of CRP, the risk of cardiac injury doubled, strongly associating systemic inflammation with airflow obstruction and the development of heart disease [57]. Another cross-sectional study that evaluated approximately 8000 adults from the ages of 20–80 found an independent association between CRP and poorer lung function defined by reduced FEV_1_. The results showed that amplified inflammatory markers were associated with lower FEV_1_ [58].

This relationship between airflow obstruction due to pressure overload, inflammation, and HF explains why chronic obstructive pulmonary disease (COPD), a common lung disease characterized by lung inflammation, is strongly associated with an elevated risk of HF [59], as demonstrated in Figure 1. Mechanistically, COPD induces chronic inflammation that damages lung tissue and peripheral airways, leading to airflow limitation and obstruction. This causes chronic inflammation throughout the body. Myocardial inflammation specifically leads to scarring of the heart muscle, or myocardial fibrosis, impacting the heart’s ability to pump, conduct electrical signals, and regulate blood flow, increasing the risk of CHF and AHF incidence in patients with COPD [60]. COPD is a risk factor for atherosclerosis, a chronic inflammatory disease that causes plaque to build up in the walls of the heart’s arteries, narrowing them and reducing blood flow. Conditions like atherosclerosis elevate the risk of ischemic heart disease, stroke, and sudden cardiac deaths in COPD patients by up to 2–3-fold [61]. TNF-α, or Tumor Necrosis Alpha, is a proinflammatory cytokine initially discovered in COPD that is a driving force behind the chronic inflammatory process of the cardiopulmonary continuum [7]. TNF-α has been associated with the severity of CHF and has been demonstrated to increase CRP levels directly, exerting adverse effects in airway obstruction, worsening COPD and increasing the risk of AHF [62].

In patients with CAD-induced CHF who also have concurrent COPD, the heart-lung interaction mechanisms play a pivotal role in disease progression. Chronic systemic inflammation, driven by CRP and TNF-α, creates a bidirectional link between cardiac and pulmonary dysfunction, where each system exacerbates the other’s decline [7,16]. For instance, cardiac dysfunction increases pulmonary pressures, leading to respiratory symptoms, while hypoxia resulting from COPD-induced airflow limitation contributes to pulmonary hypertension, further straining the heart [63–65]. These intertwined mechanisms underscore the critical impact of the heart-lung interface on overall disease outcomes in patients with combined heart and lung conditions.

In one comprehensive review study, 37 overlapping differentially expressed genes (DEGs) were found in both HF and COPD, highlighting the importance of the extracellular matrix (ECM) in both diseases [66]. The extracellular matrix (ECM) is a network of macromolecules that provides structural support to cells and tissues, regulating cellular processes through changes in its structure or biochemical composition [67]. ECM changes contribute to disease development by influencing cellular signaling pathways. The nature of both HF and COPD impacts the structure of the ECM. In HF, mechanical stress from pressure overload leads to ECM remodeling and fibrosis or tissue scarring [68]. In COPD, mechanical stress from airflow obstruction and increased lung pressure can contribute to ECM degradation [69]. Additionally, changes in ECM stiffness can affect cell behavior in both the heart and lungs. In HF, stiffened ECM can impair cardiac contractility and promote further fibrosis, often leading to AHF in the form of myocardial infarction [68]. ECM imbalances caused by the degradation of components such as elastin and collagen cause lung stiffness and airway obstruction [70]. ECM dysregulation involves common molecular pathways in both HF and COPD, where inflammatory cells are recruited to the site of injury or disease. These cells secrete cytokines and inflammatory biomarkers, such as CRP and TNF-α, which further modify the ECM and contribute to disease progression [66]. Chronic systemic inflammation as a mechanism can be further examined via ECM remodeling and degradation (Figure 1).

Oxidative stress is another link between heart and lung disease characterized by chronic systemic inflammation. Oxidative stress is caused by a chemical imbalance between producing and accumulating oxygen reactive species (ROS). Increased ROS production has harmful effects on important cellular structures like proteins, lipids, and nucleic acids [71]. Research has found that oxidative stress is responsible for diseases like atherosclerosis and should be considered a primary cause of many heart conditions [72]. Peroxynitrite, a reactive oxidant produced from ROS nitric oxide and superoxide, is the main pathogenic mechanism for AHF conditions such as stroke, myocardial infarction, and CHF [73]. Research has demonstrated that obstructive lung diseases such as asthma and COPD are linked to oxidative stress, as ROS enhances inflammation by activating kinases involving inflammatory biomarkers [74].

Another related mechanism is endothelial dysfunction, which occurs when the inner lining of blood vessels (the endothelium) is unable to maintain vessel relaxation through the production of nitric oxide. An over or underproduction of nitric oxide creates an imbalance between the factors that relax and contract vessels, narrowing the arteries [75]. Endothelial dysfunction results in a chronic inflammatory process, elevating the risk of cardiovascular events. Endothelial dysfunction has also been associated with elevated CRP and chronic systemic inflammation [76]. As mentioned above, elevated levels of CRP are associated with excess mortality in cardiovascular disease and stroke. They are often linked specifically with CHF, aortic valve disease, and AHF conditions like myocardial infarction and myocardial fibrosis [58], as well as restrictive lung diseases [59]. Therefore, oxidative stress and endothelial dysfunction prove that chronic inflammation is a likely link between heart and lung function (Figure 1).

When discussing inflammation as a risk for cardiovascular and lung disease, one must discuss smoking, as it is the main risk for atherosclerotic cardiovascular disease [77]. Smoking cigarettes increases the number of macrophages, or immune cells, in the lungs and bronchoalveolar areas, increasing the severity of inflammation and airflow limitation, and thus the severity of lung conditions like COPD. Although macrophages are immune cells, smoking impairs the function of the immune system and its ability to combat foreign invaders which is an important contributor to lung disease progression from smoking [78]. Another mechanism of cigarette smoke’s effect on the lungs is its induction of apoptosis in macrophages, leading to mitochondrial damage and oxidative stress [79]. Overall, smoking is considered a link between airway inflammation, chronic systemic inflammation, and the progressive development of atherosclerosis. One study found that smoking is linked to a higher risk of poorer clinical outcomes after AHF ischemic stroke [80]. Smoking causes a chronic inflammatory state by raising levels of inflammatory biomarkers known to be indicators of CHF/AHF events and lung diseases such as COPD.

In addition to smoking, exposure to environmental pollutants, such as particulate matter from air pollution, has been shown to significantly worsen lung inflammation and increase the risk for both COPD exacerbations and acute heart failure via systemic inflammation. Airborne pollutants, such as nitrogen dioxide (NO_2_), ozone (O_3_), and fine particulate matter (PM_2.5_), are associated with increased lung oxidative stress and inflammation. Studies have shown that air pollution increases levels of inflammatory biomarkers, such as CRP and TNF-α, which are linked to the worsening of both heart and lung diseases. Furthermore, long-term exposure to polluted air increases the likelihood of cardiovascular and pulmonary morbidity, including exacerbations of chronic heart failure and COPD [81].

Biological sex may affect how inflammatory conditions like atherosclerosis develop and progress. Men have more inflammatory plaque associated with a higher level of macrophages than women of the same age and are more likely to have plaque bleeding. Aging can exacerbate inflammation. The term “inflamm-aging”, coined by Claudio Franceschi, describes an age-related disorder characterized by increased levels of inflammatory markers such as hsCRP (high-sensitivity CRP) [82]. One study found that women aged 51–60 have higher levels of inflammation markers than men. As estrogen levels drop after menopause, women’s risk of cardiovascular disease increases [83]. Still, there is no fundamental mechanistic difference in the way that chronic systemic inflammation links HF and lung disease in terms of age and sex.

## 5. Management and Treatment

Both CHF and AHF have well established treatment and therapy plans. CHF treatment primarily focuses on the prevention of risk factors like CAD, blood pressure control, and improving lifestyle habits, such as the cessation of smoking [84]. Broadly, therapy for CHF focuses on identifying and correcting potentially reversible causes of CHF. For patients with AHF, the “time-to-treatment” approach is prioritized to lower mortality and to prevent AHF patients from developing CHF. Immediate diagnostic tests and non-pharmacologic treatment are most initiated in urgent care. For lung dysfunction and diseases such as COPD, treatment plans often focus on pulmonary rehabilitation, oxygen therapy, and inhaled medicines that open and reduce swelling in the airways, among other treatments [1]. Furthermore, minimizing exposure to environmental pollutants has been identified as a key preventative measure for HF and COPD. Reducing air pollution exposure leads to decreased possibility of exposure to inflammatory stimuli and oxidative stress, ultimately improving outcomes in cardiopulmonary diseases. Public health interventions aimed at lowering air pollution levels in urban areas have shown positive effects in reducing hospitalizations related to heart and lung diseases [85].

Although HF and lung dysfunction have historically been treated separately, the most promising joint treatment plan for heart failure and lung dysfunction involves the pharmacological management of inflammatory conditions that worsen both conditions. For example, certain drugs have been used to target heart disease and CHF, including statins, which lower cholesterol and reduce the risk of AHF incidence. Additionally, angiotensin converting enzyme (ACE) inhibitors and angiotensin receptor blockers (ARBs) target CHF by regulating blood pressure and hypertension [86]. A recent study has suggested that statins and ACE inhibitors might also improve lung conditions by reducing overall inflammation. These drugs have already proven effective for heart disease and HF patients while not worsening lung function. However, one challenge faced by co-treatment attempts is that statins, though effective in lessening inflammation, have not been proven to decrease HF morbidity significantly. Contrarian perspective may suggest that statins’ failure to improve CHF morbidity rates indicates that inflammation is not a major cause of HF. However, it could also be possible that once HF reaches a certain state, reducing inflammation cannot recover left ventricular heart function [87].

## 6. Conclusions

Both chronic and acute heart failure significantly impact lung function, leading to airway obstruction, pulmonary edema, and the exacerbation of preexisting lung conditions such as COPD. The relationship between HF and lung disease is bidirectional, as declining lung function can also exacerbate, worsen, and place patients at a higher risk of CHF and AHF due to pressure overload and inflammation. This bidirectional link has been demonstrated across a variety of clinical studies and animal trials. Heart and lung conditions are typically treated separately, but combined treatment approaches show promise. For example, statins, ACE inhibitors, and ARBs, which typically target HF, may also benefit lung conditions by reducing inflammation. Understanding the connection between the heart and lungs allows for a more integrated approach to treating both heart and lung conditions. This can lead to better patient outcomes by addressing the complex relationship between these diseases and potentially reducing the overall clinical burden of heart failure and lung disease.

## Figures and Tables

**Table 1.** Clinical evidence highlighting the association between heart failure and lung dysfunction.

Population	Study Design	Key Findings	References
CHF patients	Multicenter Cardiovascular Cross-Sectional Study	Higher COPD prevalence in CHF patients (20%) than the general population (13%).	Hawkins et al., 2009 [23]
North American and European CHF patients	Meta Analysis Review	COPD prevalence: 11–52% (North America) and 9–41% (Europe), higher in recent studies.	Silvestre et al., 2018 [24]
Community-based (~10,000 participants)	Longitudinal Cohort Study	Decline in lung function linked to increased CVD risk, independent of other factors.	Chen et al., 2015 [25]
COPD patients	Meta Analysis Review	COPD patients have a 95% higher likelihood of CVD, with 2–5 times increased risk of ischemia, arrhythmia, and CHF.	Sin et al., 2005 [26]
~84,000 participants across 12 studies	Population-Based Study & Systematic Review	Poor lung function predicts cardiovascular mortality; the lowest lung function quintile had a 75% increased risk.	Eriksson et al., 2013 [27]
642 randomly selected subjects (22–72 years)	Cross-Sectional Patient Survey Study	Strong link between ischemic heart disease and impaired lung function; COPD severity correlated with heart disease prevalence.	Zoghi et al., 2009 [29]
~6630 participants (≥50 years old)	Observational Study	COPD contributes to adverse cardiovascular events, such as myocardial infarction.	Mannino et al., 2008 [31]
~20,300 subjects (≥45 years)	Observational Study	Restrictive lung function is associated with incident cardiovascular issues.	Iqbal & Gupta, 2023 [32]
AHF/MI patients	Observational Patient Study	Myocardial infarction is a major cause of pulmonary edema.	King & Goldstein, 2023 [20]
~7000 stable CHF patients	Observational Patient Study	46% of CHF patients had moderate-to-severe sleep-disordered breathing.	Marin et al., 2005 [35]
~1650 patients with varying sleep conditions	Comparative Longitudinal Study	Untreated severe OSA increased CVD risk 3-fold compared to those without sleep-disordered breathing.	Milani-Nejad & Janssen, 2014 [36]

**Table 2.** Preclinical evidence demonstrating heart failure-induced pulmonary changes in animal models.

Study Design	Key Findings	Implications	References
Induced TAC CHF in mice	Chronic TAC caused pulmonary fibrosis, lung remodeling, pulmonary hypertension, increased lung weight, and RV hypertrophy.	Demonstrates the link between left ventricular failure and lung disease, highlighting significant lung remodeling and pulmonary hypertension.	Olver et al., 2019 [40]
TAC-induced heart failure in Ossabaw pigs	Chronic pressure overload caused heart failure symptoms, increased lung weight, and pulmonary hypertension.	Validates the TAC model in larger animals to study CHF with preserved ejection fraction and its lung impact.	Ma et al., 2019 [41]
Induced myocardial infarction in mice	MI resulted in increased lung weight, pleural effusion (60% of mice), and pulmonary edema.	Connects MI-induced heart failure with lung dysfunction, such as pleural effusion and pulmonary edema.	Andersen et al., 2023 [42]
Review of various animal trials	Animal models have limitations due to physiological differences and discrepancies with human conditions.	Highlights limitations of animal models but emphasizes their importance in developing treatments and understanding heart-lung connections.	Feldman, 2011 [43]
Review of animal drug trials	Animal trials contributed to beta blocker development, reducing stress on the heart and blood vessels.	Demonstrates the role of animal trials in developing treatments for heart failure, particularly beta blockers.	Ramalho & Shah, 2021 [44]; Cundrle et al., 2019 [45]
